# HLA-G as a Tolerogenic Molecule in Transplantation and Pregnancy

**DOI:** 10.1155/2014/297073

**Published:** 2014-07-21

**Authors:** Vera Rebmann, Fabiola da Silva Nardi, Bettina Wagner, Peter A. Horn

**Affiliations:** ^1^Institute for Transfusion Medicine, University Hospital Essen, Virchowstraße 179, 45147 Essen, Germany; ^2^CAPES Foundation, Ministry of Education of Brazil, 70.040-020 Brasília, DF, Brazil

## Abstract

HLA-G is a nonclassical HLA class I molecule. In allogeneic situations such as pregnancy or allograft transplantation, the expression of HLA-G has been related to a better acceptance of the fetus or the allograft. Thus, it seems that HLA-G is crucially involved in mechanisms shaping an allogeneic immune response into tolerance. In this contribution we focus on (i) how HLA-G is involved in transplantation and human reproduction, (ii) how HLA-G is regulated by genetic and microenvironmental factors, and (iii) how HLA-G can offer novel perspectives with respect to therapy.

## 1. Introduction

Both solid organ and hematopoietic stem cell transplantation (HSCT) represent life-saving therapies for patients with end stage organ failure or severe haematological malignancies, respectively. However, genetic incompatibilities between donor and recipient, in particular among classical human leukocyte antigen (HLA) class I (HLA-A, -B, -C) and class II (HLA-DR, -DQ, -DP) molecules, lead to a powerful alloresponse by the adaptive and/or innate immune system, which has to be controlled by immunosuppressive drugs. Despite the development of modern immunosuppressive strategies, the induction of such reactions cannot always be completely prevented, and acute or chronic rejection remains a major complication in transplantation. Another set of problems in transplantation arose due to the toxicity of immunosuppressive drugs. Thus, the success of transplantation depends on the balance between rejection and the side effects of modern immune suppressants.

The development of a certain degree of immune tolerance against allogeneic antigens can favour a successful outcome. In solid organ transplantation the induction of tolerance can diminish the risk of acute and chronic graft rejection and thereby improve the survival of the allograft. In HSCT tolerance may weaken host versus graft (HvG) as well as graft versus host disease (GvHD). Experimental research on naturally occurring mediators for immune tolerance represents one approach to design new strategies providing a more effective therapy of transplanted recipients.

Decades of research have identified HLA-G as a naturally occurring tolerance-inducing molecule. This molecule is operative in pregnancy, which is the only true physiological situation of tolerance towards a semiallograft. HLA-G belongs to nonclassical HLA class I family. Although it shares some structural similarities with classical HLA class I, several important differences render HLA-G unique among HLA class I molecules: it displays a low allelic variation, a restricted peptide repertoire [1–3], an unusually high diversity of molecular structures due to alternative splicing of the primary transcript [4–6], and a restricted expression under physiological conditions, which can be upregulated in various situations. HLA-G has originally been discovered on the extravillous cytotrophoblast at the maternal-fetal interface [7]. It is also expressed by amnion epithelial cells [8, 9], erythroid, and endothelial cells of fetal blood vessels in the placenta [10, 11], as well as in the thymus [12], cornea [13], pancreas [14], and nail matrix [15]. Although marginal, the levels of HLA-G specific transcripts are found in nearly all tissues analysed, for example, fetal liver, myelomonocytic cells, fetal and adult eye tissue, skin and keratinocytes, and peripheral blood lymphocytes [16]. The latter can release substantial amounts of soluble HLA-G (sHLA-G) into the blood circulation [17–19].

The initial discovery of HLA-G on the extravillous cytotrophoblast soon led to the concept that it is involved in mechanisms of tolerance. Today, it is seems that an enhanced HLA-G expression in transplants or in the circulation of its recipient is associated with the acceptance of allogeneic graft, but only a minority of patients express high levels of this molecule. In this contribution we focus on (i) how HLA-G is involved in human reproduction and transplantation, (ii) how HLA-G is regulated by genetic and micro-environmental factors, and (iii) how HLA-G offers novel therapeutic options in transplantation.

## 2. The Structural Basis for the Recognition of HLA-G by Immune Receptors

Seven different isoforms derived from alternative splicing of the primary transcript are known. Four of them are membrane-anchored molecules (HLA-G1, -G2, -G3, and -G4) and the remaining three isoforms (HLA-G5, -G6, and -G7) represent secreted molecules as the transmembrane region is missing [4–6]. The HLA-G1 and HLA-G5 molecules represent the full extracellular length composed of three alpha domains assembled with *β*2-microglobulin (*β*2m) in the endoplasmatic reticulum. Variants of these molecules can also exist in non-*β*2m-associated structures [20, 21]. The structures associated with *β*2m present nonameric peptides [2]. However, the peptide repertoire is restricted compared to classical HLA molecules, as all amino acid exchanges of HLA-G occur outside the peptide-binding groove of the alpha 1- and alpha 2-domain [22]. Consequently HLA-G is not recognized as a foreign antigen by the T cell receptor and therefore does not induce a T cell based immune response, which is the main functional arm in transplantation. The other non-*β*2m associated structures are truncated isoforms missing one or two *α*-domains. All full-length isoforms [23, 24] as well as all other truncated ones [25] are able to form disulphide-linked dimers due to the cysteine residue at position 42. All membrane-anchored molecules can further be released into circulation either as shed molecules [17] or via microvesicles (MV) such as exosomes [26, 27].

Regarding function, HLA-G preferentially acts as ligand for two inhibitory receptors including the immunoglobulin-like transcript (ILT) receptor-2 (LILRB1/CD85j) and ILT4 (LILRB2/CD85d). Both receptors recognize the alpha-3 domain of HLA-G, but these two receptors distinguish different structures of HLA-G: the ILT2 receptor interacts with *β*2m-associated HLA-G molecules, whereas the ILT4 receptor recognizes non-*β*2m-associated structures. ILT2/4 receptors are expressed on subpopulations of T, B, and NK cells and on monocytes/macrophages/dendritic cells [28–33]. Due to the functionality of these receptors and their expression profile, HLA-G has the capability to modulate the immune response of the adaptive and/or of the innate immune system. As sHLA-G and membrane-anchored structures exhibit the same receptor specificity, both HLA-G and its soluble counterparts are potent molecules modulating the innate and adaptive immune response. However, dimers of HLA-G/sHLA-G bind to these receptors with a superior avidity than HLA-G/sHLA-G monomers or classical HLA class I molecules do [24, 34]. This emerges the question whether the generation and/or the quantity of dimers may regulate the signaling potential of these ILT receptors. Receptor binding assays, enabling to define the amount of dimers as recently introduced [35], may give further lights into this problem.

HLA-G is also reported to be ligand for the killer immunoglobulin-like receptor 2DL4 (KIR2DL4/CD158d), which is expressed on NK cells [36]. Remarkably this receptor is mainly expressed on decidual NK cells, suggesting a delicate role in pregnancy [29]. At variance to ILT2/4, the monomeric/dimeric status of HLA-G has no impact on the binding avidity towards this receptor [23, 24, 34]. Even more, the functional outcome of HLA-G/KIR2DL4 engagement is not easily predictable in an in vivo situation, as KIR2DL4 bears two elements, the one for inhibition in its cytoplasmic tail and the other one for activation in the transmembrane region. Furthermore, controversial information about the surface expression of 2DL4 on NK cells and its exclusively and specific binding to HLA-G as well as the resulting functional consequence are available: in early studies the surface expression of this receptor on peripheral blood cells was found to be expressed in every human NK cell clone tested and in all human subjects [29], whereas later studies demonstrated that KIR2DL4 expression depends on certain KIR2DL4 alleles [37] and is normally restricted to a CD56high subset of NK cells [38] even if KIR2DL4 is constitutively expressed on a transcriptional level in all NK cells [39]. Clearly, KIR2DL4-immunoglobulin fusion protein is able to interact with surface expressed HLA-G or soluble forms [29, 40, 41]. Here it is reported that residues Met76 and Gln79 specific for HLA-G are involved in KIR2DL4 recognition [36]. As 2DL4 is found to be present in endosomes of resting NK cells colocalized with sHLA-G, it was suggested that sHLA-G molecules were internalized after binding to 2DL4 [41]. Functionally, KIR2DL4 is reported to trigger a potent proinflammatory production of cytokines, chemokines, and angiogenic factors but only weak cytotoxicity in resting NK cells [38, 41, 42]. Whether the functional consequences are exclusively attributed to the interaction of KIR2DL4 to HLA-G is a matter of debate as the first results on this topic were partly corrected by the authors themselves as followed: the expression system in the cell line NK-92 that was used to test functional recognition of HLA-G by KIR2DL4 had been unreliable as the NK-92 cell line infected with recombinant vaccinia viruses that do not encode KIR2DL4 showed the same results. Recently, it was figured out that the IFN-*γ* response of freshly isolated NK cells towards soluble HLA-G preparations was found to be absolutely dependent on the presence of small numbers of contaminating myeloid dendritic cells. This leads to the question whether the detected cytokine response mentioned in other studies might be due to improper NK cell isolation and due to the availability of other cell populations bearing HLA-G specific receptors such as myeloid dendritic cells [43]. Moreover, KIR2DL4 can interact with alternative ligand(s) expressed by cells of epithelial or fibroblast origin. Importantly, KIR2DL4 recognition of cellular ligands is directly regulated by heparan sulfate glucosamine 3-O-sulfotransferase 3B1 [44]. Thus, studies on the interaction of cell-surfaced expressed KIR2DL4 and HLA-G should be critical reevaluated with regard to the receptor specificity and the presence of heparan sulfate glucosamine 3-O-sulfotransferase 3B1.

Similar to classical HLA class I molecules the full extracellular length HLA-G structures (HLA-G1 and HLA-G5) are able to interact with the CD8 coreceptor on T and on subpopulations of NK cells. Here the affinity to CD8 does not differ between HLA-G and classical HLA class I molecules [45]. Notably, the engagement of sHLA-G1/G5 with CD8 results in the induction of Fas/FasL-mediated apoptosis in CD8 bearing cells and in the inhibition of cytotoxic T cell activity [46–48].

Furthermore, HLA-G molecules can indirectly interact with the activators CD94/NKG2C and inhibitory CD94/NKG2A receptor/ligand system. Here, a nonameric peptide derived from leader sequence of HLA-G seems to increase and stabilize HLA-E, another member of the nonclassical HLA class I family at the cell surface [49]. These receptor systems are expressed on NK cells and on certain subpopulations of other effector cells [29, 50].

## 3. How Can HLA-G Shape the Immune System towards Tolerance in Transplantation and Pregnancy?

In transplantation and pregnancy, tolerance can be achieved by processes of deletion of reactive cells, by induction of anergy, and by other immune regulating mechanisms like the induction of regulatory/suppressor cells. Regulatory cells may exist in various cell subpopulations including antigen presenting cells (APC), CD8 T cells, and CD4 T cells.

**Figure 1:**
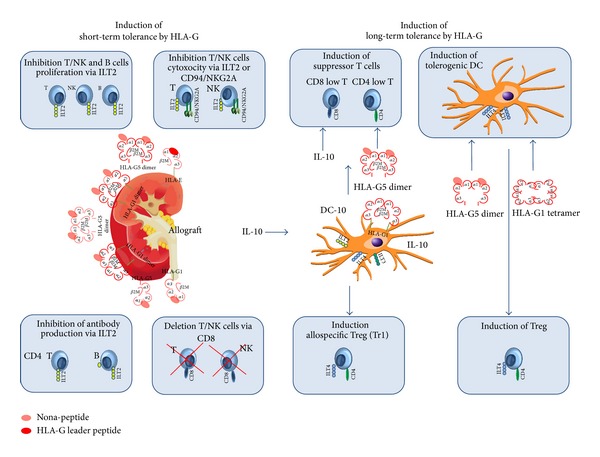
Induction of short- and long-term tolerance by HLA-G. Short-term tolerance will be achieved by HLA-G via interaction of allograft derived monomeric or dimeric *β*2-associated HLA-G1 and HLA-G5 molecules with the ILT2 receptor on T, NK, and B cells resulting in the inhibition of cytotoxicity, proliferation, or antibody production. Short-term tolerance can be indirectly induced by HLA-G via the presentation of HLA-G specific leader peptide through HLA-E and its interaction with the cognate inhibitory receptor heterodimer CD94 and NKG2A on T and NK cells subsets. The interaction of HLA-G5 with CD8 coreceptor on certain T and NK cell population leads to the deletion of these cells. Long-term tolerance will be achieved by the induction of different types of regulatory T (Treg) cells. Allospecific Treg (Tr1) will be generated by so-called DC-10 cells expressing ILT2, 3, 4 receptors together with *β*2-free or -associated HLA-G1 via a ILT4/HLA-G pathway. Suppressor T cells expressing low CD4 or CD8 coreceptors are induced in the presence of HLA-G5 molecules together with IL-10 or HLA-G positive antigen presenting cells. The interaction of non-*β*2-associated HLA-G5 dimers or HLA-G1 tetramers with ILT4 antigen presenting cells results in the induction of tolerogenic DC, which favours the induction of Tregs.

In vitro experiments have shown (Figure 1) that HLA-G/sHLA-G molecules derived from transplanted patients or from isolated cultured cytotrophoblast cells initiate a short-term inhibitory effect on T cells, NK cells, and B cells by binding with ILT2, ILT4, and KIR2DL4 [51–54]. In consequence, HLA-G inhibits the activity of CD8 positive cytotoxic T cells, peripheral and decidual NK cells [46, 48, 55–57], the allogeneic proliferation response of CD4 positive T cells [58] and cell cycle progression of alloreactive T cells [59, 60]. In all experimental settings the inhibitory effect of HLA-G could be abolished in the presence of a blocking antibody specific for HLA-G. Interestingly, the binding of HLA-G with ILT2 suppresses both, the naive and the memory B cell functions. In particular HLA-G is able to impede B cell proliferation, differentiation, and Ig secretion in both T cell-dependent and -independent models of B cell activation. Furthermore, it seems that HLA-G acts as a negative B cell regulator in modulating B cell antibody secretion in a xenograft mouse model [61].

An indirect inhibitory effect of HLA-G is that its presence leads to an enhanced expression of ILT2, ILT3, ILT4, and KIR2DL4 receptors in antigen-presenting cells, NK cells, and T cells. This upregulation seems not to require any antigenic costimulation. It is discussed that by the upregulation of inhibitory receptors the threshold level for an immune activation is increased in effector cells of recipients, which in turn may result in a better graft acceptance [53]. A further important indirect way, how HLA-G exerts inhibition of effector function, is the induction of HLA-E on cell surface expression by providing HLA-G specific leader peptides. The interaction of HLA-G leader peptides with the inhibitory receptor complex CD94/NKG2A results in prevention of NK cell-dependent lysis. Lastly, HLA-G-expressing cells modulate the ability of decidual mononuclear cells or peripheral blood mononuclear cells to release cytokines in a way that may shift the Th1/Th2 balance towards relative Th2 dominance [62]. In addition, the presence of sHLA-G molecules stimulates especially the release of IL-10 [63].

In addition to these short term effects (Figure 1), HLA-G can also provide long term inhibitory effects by the induction of regulatory or suppressor cells [52] that should contribute to the development of tolerance. The interaction of HLA-G with the ILT4 receptor can initiate such long term effects. In a mouse transplant model [64, 65] it has been shown that the engagement of ILT4 with HLA-G5 dimer or HLA-G1 tetrameric complex on antigen presenting cells (APC), such as dendritic cells (DC), inhibits their maturation resulting in anergy and in a diminished MHC class II, CD80, and CD86 expression. These anergic DCs have the capability to favour the differentiation of regulatory T cells and to prolong significantly allograft survival. In addition there is first evidence that myeloid cells are also sensitive to HLA-G, as they have ILT2 and ILT4 receptors expressed on cell surface. In ILT2 transgenic mouse model it could be demonstrated that HLA-G induces the differentiation of myeloid derived suppressor cells that promote long term graft survival [66].

An indirect inhibitory effect of HLA-G, which induces long term tolerance, has been shown for CD4+T cells and CD8+T cells stimulated by HLA-G1-expressing APC [67]. These T cells lose their capability to response to stimulation and differentiate into regulatory T cells. Once generated, the tolerance-inducing functions of these cells are independent from HLA-G. HLA-G-expressing APC can be generated in vitro by the differentiation of monocytes into DC in the presence of interleukin-10 (IL-10) [68]. These so-called DC-10 are tolerogenic DCs and characterized by the high expression intensities of HLA-G1, ILT2, ILT4, and ILT3 and their production of high IL-10 levels. Functionally, DC-10 is able to induce adaptive allospecific regulatory T cells (Tr1) through the IL-10-dependent ILT4/HLA-G pathway. Tr1 cells are known to exert immune suppression by producing high levels of IL-10 in the absence of IL-4 [68, 69]. Such DC-10 can also be found in vivo in peripheral blood as well as in secondary lymphoid organs. In the pregnancy microenvironment, the maintenance of tolerance during the first trimester may be one of the major roles of this APC subset. The human decidua presents higher frequency of tolerogenic DC-10 if compared with peripheral blood of pregnant women. This observation can be attributed either (i) to the recruitment of DC-10 from peripheral blood or (ii) to the conversion of resident decidual DCs into DC-10 or lastly (iii) to* de novo* induction of DC-10 by the IL-10 dependent ILT4/HLA-G pathway [70].

## 4. How Is HLA-G Expressed in the Normal or Pathological Course of Pregnancy or Transplantation?

Most of the functional implications of HLA-G in tolerance have been established in in vitro models. However, studies on HLA-G expression in allogeneic situations such as pregnancy and transplantation and its association to the clinical course further support the crucial role of HLA-G in mediating or maintaining tolerance in vivo.

## 5. HLA-G Expression in Pregnancy

HLA-G specific transcripts are detectable in oocytes as well during implantation development: The frequencies of embryos expressing the full extracellular length isoforms HLA-G1/G5 increase with ascending developmental and cleavage stages [71, 72], and the presence of HLA-G transcripts in blastocysts is associated with higher cleavage rate and a higher pregnancy rate [73, 74]. Several single and multicentre studies (for review see [75–77]) have firmly demonstrated that sHLA-G molecules can be found in embryo cultures (EC) after human-assisted reproduction techniques (ART). In concordance to the mRNA expression profile the proportion of sHLA-G positive EC increases with the developmental stages of the embryo [78], indicating that besides the morphological scoring system of embryos the detection of sHLA-G in EC represents an additional parameter for the prediction of pregnancy outcome after ART. With regard to HLA-G structures there are two studies demonstrating that only half of HLA-G-expressing blastocysts coexpressed the *β*2-m transcripts [73, 74]. This indicates that these blastocysts have the prerequisite available to form functional active *β*2m-associated HLA-G1 dimers known to interact with ILT2, whereas the other HLA-G-expressing blastocyst should at least be qualified to form non-*β*2m-associated structures interacting with the ILT4 receptor. Interestingly, the alternatively spliced transcripts HLA-G3 and HLA-G4 isoforms are the most abundant ones during implantation development [71]. This observation raises questions about the functional relevance and possible novel unknown HLA-G receptors for HLA-G3/4 operative in pregnancy, which may also be relevant in transplantation.

The arrival of the blastocyst in the uterine cavity promotes functional and molecular immune modulatory changes in endometrial cells and in the blastocyst itself, resulting in adhesion, trophoblastic invasion, decidualization, and placentation [76]. Taking into account all the mechanisms leading to a successful embryo implantation, the unusual phenotype of the semiallogeneic extravillous trophoblastic cells (ETC) seems to have the most prominent influence on the induction of feto-maternal tolerance. The ETC preferentially express HLA-G (for overview see [75, 76]) as well as low levels of HLA-E and HLA-F [79], a third nonclassical HLA molecules, but do not express classical HLA class I (HLA-A and -B) and HLA class II (HLA-DR, -DQ and -DP). HLA-G is recognized to be a key molecule in embryo implantation, modulating the local immune response by mechanisms mentioned before, which in sum results in the suppression of the maternal immune system (for overview see [75, 80, 81]). In normal pregnancy the membrane-bound HLA-G1 and the secreted HLA-G5 isoforms are the most abundant HLA-G molecules during invasions. Interestingly, different structures are found in certain trophoblast subpopulations: At the leading edge of trophoblast columns and on invading ETC HLA-G1 isoform is expressed as *β*2-associated homodimer [82]. The HLA-G5 isoform is ubiquitously expressed in all types of trophoblast populations either in a *β*2-associated structure on ETC or in a non-*β*2m-associated dimeric structure on villous trophoblasts [83]. HLA-G5 produced locally by ETC is discussed to alter the maternal cytokines profile in a normal successful pregnancy. Recently, a multicenter study has provided substantial evidence [84] that the HLA-G5 can induce the IL-4 production by decidual CD4+T cells via ILT2 receptor expression on decidual T cells. Here, ILT2 seems to be upregulated on the activated CD4+T cells and downregulated in activated macrophages. The increased number of ILT2 receptors on the CD4+T cell surfaces enhances the binding efficiency of the HLA-G5 molecule and consequently the secretion of IL-4, which appears to be one of the critical anti-inflammatory interleukins for successful pregnancy.

Interestingly, CD4 or CD8 positive T cells expressing constitutively HLA-G can also be found in the peripheral blood and in the decidua during pregnancy [70]. These cells are described to exert T regulatory (Treg) functions in vitro like the potent suppression of T cell proliferation. Originally, these kinds of Treg cells have been discovered to be accumulated at site of inflammation [85].

Recurrent spontaneous abortion (RSA) and preeclampsia have been discussed to be caused by an insufficient immune tolerance at the maternal-fetal interface. Considering the functionality of HLA-G, the low HLA-G expression in placental tissue obtained from women experienced pregnancy complications supports these discussions [86–89].

During the course of an uncomplicated pregnancy altering sHLA-G levels are observed in the peripheral blood of the mother's levels: in the first gestational trimester the sHLA-G levels increase until reaching the highest peak at month three of gestation [90, 91] and start to decrease again during the third trimester [92]. Undetectable sHLA-G or variation in the course of sHLA-G especially during in the first weeks of gestation seems to be related with gestational complications, implantation failure, miscarriage and spontaneous abortion, preeclampsia, and placental rupture (for a concise review see [75, 76]). It has been reported that sHLA-G molecules contribute to modulation of maternal cytokine profile, in particular the upregulation of anti-inflammatory cytokines such as IL-3, IL-4, and IL-10 [93].

## 6. HLA-G and Transplantation

The first evidence that HLA-G expression is involved in transplantation has been reported for heart transplantations [94]. In 36 noninflammatory heart transplant recipients, the expression of HLA-G in endomyocardial biopsies or in serum samples of patients had clearly been associated with a decrease of acute and chronic cellular rejection episodes [94]. These results have been confirmed by other studies indicating that HLA-G or sHLA-G molecules contribute to an improved heart allograft acceptance after transplantation [95, 96]. Regarding sHLA-G, both the basal levels in pretransplant patients and its alteration after heart transplantation seem to be useful to monitor the development of transplantation. Interestingly, a recent study provides first hints that in patients with a biopsy proven humoral rejections the circulating sHLA-G levels are reduced compared to patients without any sign of rejections [97]. Here, it is very likely that sHLA-G is involved in the modulating B cell functions in vivo, as recently evidenced by in vitro experiments [61]. Regarding the source of sHLA-G, one in vitro study gives hints that during an allogeneic reaction HLA-G5 is released by allospecific CD4(+) T cells, resulting in the suppression of the proliferative T cell response [98]. Controversial results have been reported for cardiac allograft vasculopathy (CAV), which is a major limitation of long-term survival after heart transplantation: in one study sHLA-G levels have been found to be increased in recipients without heart allograft CAV compared to recipients suffering from severe CAV post transplantation [99], whereas the another one could not observe any differences between these two patients groups [100].

In lung, liver, kidney, combined liver-kidney, or combined kidney-pancreas transplantation there is an overall concurrence that increased HLA-G expression in biopsies [101–103] or high circulating sHLA-G levels [102, 104–108] has always been associated with a better graft acceptance in terms of acute and chronic cellular rejections. Of note, in kidney transplant patients, there is an inverse association of sHLA-G with organ failure from chronic rejection and with the production of anti-HLA IgG antibodies. This is the second evidence that HLA-G contributes to the modulation of B cell functions in vivo, which in turn might prevent a humoral rejection. In kidney, liver, or combined liver-kidney transplantation, enhanced sHLA-G levels are concomitantly with high IL-10 levels and novel CD3+CD8low and CD3+CD4low suppressor T cells being negative for FOXP3, a common marker for regulatory T cells. In vitro experiments (Figure 1) have shown that these two types of regulatory T cells can be induced by HLA-G-expressing APC or HLA-G5 molecules [104].

Several studies have analysed the putative role of HLA-G/sHLA-G expression in the allogeneic hematopoietic cell transplantation setting. Indeed, increased HLA-G5 levels on day 15 and on day 30 after HCST seem to be negatively correlated with the severity of aGvHD [109] suggesting HLA-G5 as a predictor of the occurrence and severity of aGvHD. This concept has been further sustained by the fact that high levels of HLA-G before and after allograft are indicative for good HCST outcome with respect to the occurrence of aGvHD. The involvement of sHLA-G molecules in aGvHD prevention is further supported by the positive correlation of sHLA-G levels with the number of regulatory T cells [110]. Nevertheless, in the study of Waterhouse et al. [111] certain sHLA-G levels could not be related to aGvHD, disease recurrence, or death implying a weak or negligible involvement of sHLA-G in the outcome of HCST.

## 7. The Impact of the Genetic Background on HLA-G Expression in Pregnancy and Transplantation

In general,* HLA-G* presents low polymorphisms as compared to classical HLA class-I antigens. The IMGT/HLA Database (http://hla.alleles.org/, version 3.14.0, October 2013) contains fifty* HLA-G* alleles resulting in 16 different proteins (HLA-G∗0101, ∗0102, ∗0103, ∗0104, ∗0106, ∗0107, ∗0108, ∗0109, ∗0110, ∗0111, ∗0112, ∗0114, ∗0115, ∗0116, ∗0117, and ∗0118) and 2 null alleles. The variations in the amino acid sequence between the allelic variants of* HLA-G* are located in coding and in non-coding regions, which may affect the biological properties of HLA-G.

In the coding region, 18 out of 50 HLA-G alleles determine four membrane-bound (HLA-G1, HLA-G2, HLA-G3, and HLA-G4) and three soluble forms of HLA-G (HLA-G5, HLA-G6, and HLA-G7) produced by alternative splicing. The exceptions are represented by currently two described null alleles (HLA-G∗01:05N and HLA-G∗01:13N), which lead to the generation of a reduced number of HLA-G isoforms or to the lack of a functional protein. The HLA-G∗01:05N allele is the result of a deletion at position 130 in exon 3 leading to a shift in the open reading frame and the generating of a stop codon (TGA) at position 189 in exon 4 [112]. In consequence, the synthesis of the *α*2 domain is prevented. The exclusive synthesis of HLA-G2, -G3, and -G7 isoforms remains to imply an immune relevant property of this structural variants [113]. In HLA-G∗01:13N a single exchange of cytosine to thymidine at position 54 results in a stop codon (TAG) in exon 2 and in the absence of a HLA-G protein [114]. The frequency of HLA-G∗01:05N is higher than 10% in African and Indian populations [115, 116], whereas the null allele HLA-G∗01:13N is rare and has only been described in the African population [114].

Four variable amino acid positions are present in the *α*1 domain (codon 13, 27, 31, and 54), six variations in the *α*2 domain (100, 104, 105, 110, 130, 159, and 169), and four in the *α*3 domain (185, 189, 219, and 258) [117–119]. These variations may lead to altered protein conformation of HLA-G, which could result in different biological functions, for example, affinity to receptors. Three different alleles have been described to vary in the intron 2, 3 and 5 regions [120–122]. The underlying importance and mechanism are unknown, yet.

Due to the tolerance-mediating role of HLA-G great efforts have been made to identify genetic factors influencing the HLA-G expression to predict its presence in allogeneic situations and thereby to estimate the development of naturally occurring immune tolerance.

The concept that HLA-G expression is under genetic control has been introduced by the observation that the amounts of circulating sHLA-G molecules are associated to certain HLA-G alleles [123]. In this study high levels could be related to the* HLA-G*∗*01:04:01,* whereas reduced sHLA-G levels has been associated with the alleles* HLA-G*∗*01:01:03* and* G*∗*01:05N*. Whereas* HLA-G*∗*01:04:01 *and* HLA-G*∗*01:01:03* represent synonymous nucleotide variations in the coding region compared to the most common allele* HLA-G*∗*01:01:01*, the HLA-G∗01:05N is defined by the loss of HLA-G1 expression as well as its soluble counterpart HLA-G5 as already mentioned. Interestingly, these alleles are often reported to be associated in unexplained recurrent abortion [116, 123–125]. In addition, these HLA-G alleles could be related to a higher rejections probability in renal transplant patients [126].

The noncoding region of the* HLA-G* gene consists of the promoter region and the 3′UTR region, which are important for the regulation of gene expression and mRNA stability. The majority of polymorphic sites is located in the promoter 5′ upstream regulatory (5′URR) and 3′ untranslated (3′UTR) regions have been reported to influence HLA-G expression [127, 128]. Regarding to the 5′URR promoter region [129, 130], the unusual feature of the cis-acting regulatory elements, such as the modified enhancer A (enhA) and a deleted interferon-stimulated response element (ISRE), modifies its binding properties and determine its responsiveness to nuclear factor-kappa B (NF-*κ*B) and interferon-*γ* (IFN-*γ*), which in turn can influence the protein expression of HLA-G. To date 29 SNP positions have been identified, all of which flank promoter elements [131, 132]. Clinically relevant polymorphisms in the 5′URR are scarce: the A/C SNP at position −486 and the 725G allele have been found to be associated with miscarriage [133, 134]. The presence of a guanine at position −725 creates a CpG dinucleotide between −725 and −726 nucleotides, which can be methylated on −725G variants. This epigenetic variance may influence the binding site for the transcription factor IRF-1 (interferon regulatory factor-1) and interfere in HLA-G transcription and consequently in HLA-G expression [133]. Furthermore, SNPs at positions −964 G/A, −725 C/G or C/T and −486 A/C are reported to be linked with an increased risk to develop an acute kidney allograft rejection being observed [135].

The 3′UTR region of the* HLA-G* gene is located distal of a stop codon in exon 6 meaning that exons 7 and 8 are not translated and missing in the mature mRNA. This region includes regulatory elements including AU-rich motifs and polyadenylation signals; and three important polymorphisms have been associated with the regulation of HLA-G expression.

First, the 14 bp fragment (5′-ATTTGTTCATGCCT-3′) insertion or deletion has been associated with differences in the stability of the HLA-G mRNA [136]. The presence of the 14 bp insertion is related to increased stability of the transcript and with mechanisms of posttranscriptional control (alternative splicing), whereby 92 bases from the mature transcript are removed from the start of exon 8 [137]. Furthermore, there is the possibility of linkage disequilibrium (LD) among polymorphic sites in the 3′UTR with polymorphic sites in the 5′URR may leading to a different posttranscriptional patterns and changes in the HLA-G expression [128, 137, 138]. Importantly, mRNA from 14 bp insertion shows an increased surface expression of HLA-G1 expression relative to mRNA lacking the 14 bp sequence but the ratio of release sHLA-G1 to surface expressed HLA-G1 is lower [139]. In line with this, low sHLA-G levels are often associated with the 14 bp insertion genotype (+14/+14 bp) in women having experienced pregnancy complications [140–143]. On the other hand in healthy individuals [144] and in heart transplantation, the highest production of sHLA-G has been related to the 14 bp deletion/deletion genotype in pre- and posttransplantation resulting in low rejection rates. In addition, the 14 bp polymorphism of the* HLA-G* gene is related to the cyclosporine absorption of the patient [145, 146].

In bone marrow transplantation, although there are considerable evidences regarding the immunomodulation role of the HLA-G, only a few studies have evaluated the polymorphic sites in the HLA-G gene, particularly the 14 bp INDEL polymorphisms in the 3′UTR region. The 14 bp deletion homozygous genotype (−14/−14 bp) is associated with severe aGvHD, while the insertion homozygous genotype (+14/+14 bp) and the heterozygous genotype (+14/−14 bp) are associated with low risk for aGvHD in bone marrow transplantation for thalassemia patients [147]. Moreover, in HSCT recipients for hematologic malignancies, the homozygous insertion genotype +14/+14 bp was recently found to be associated with disease-free survival [148]. In contrast, the homozygous state for the 14 bp insertion presents a risk factor for severe aGvHD (grade III and IV) after HSCT using bone marrow as stem cell source from HLA-matched donors [149]. In two other studies the 14 bp INDEL allele and genotype frequencies revealed no significant differences in allo-HSCT posttransplantation complications such as acute or chronic GvHD [111, 150].

Second, the small nucleotide polymorphism (SNP) at position +3142 C/G in the 3′UTR of the* HLA-G* gene is proposed to be a target for certain micro RNAs (miRNA), which results in degradation of the HLA-G mRNA. The presence of guanine at position +3142 may increase the affinity for miR-148a, miR-148b, and miR-152, which may downregulate the expression of HLA-G by RNA degradation or translation suppression [151, 152].

Third, a SNP at the position +3187 A/G has been described recently. The presence of the +3187A allele causes decreased HLA-G expression because of its proximity to an AU-rich element related to mRNA degradation [153, 154]. The +3187A allele may be associated with pre-eclampsia [153].

In only one study all three polymorphic sites at 3′UTR have been analysed for kidney recipients. Among them the presence of the +3187A allele gives the highest association to acute and chronic rejections after kidney transplantation, whereas the others are only of marginal relevance [155].

Attempting to clarify the contradictory findings regarding HLA-G polymorphisms and disease associations it is interesting to point out that the presence of these two cited sites of polymorphisms besides the 14-bp polymorphism (+3142 C/G and +3187 A/G), both related with low mRNA availability, can be disposed in haplotypes and are possibly acting together at the HLA-G production scenario and might lead to different posttranscriptional patterns and changes in the HLA-G expression [132].

In conclusion, the impact of the genetic background on HLA-G expression in pregnancy and transplantation remains an open question. Further systematically evaluation of polymorphic sites in 5′URR and 3′UTR and their relationship to the HLA-G/sHLA expression in allogeneic situation have to be performed to understand how HLA-G posttranscriptional mechanisms may contribute to tolerance. Under the assumption that the HLA-G expression is preferentially controlled by genetic factors, in transplantation the genetic background of both the donor and the recipient has to be taken into account. The donor type is of extraordinary importance as it should mirror the HLA-G expression in the graft, where the acute/chronic rejection actually takes place in solid organ transplantation. The genetic background of the recipient may control the expression by the recipient's effector cells for example, HLA-G expression on APC or release of sHLA-G molecules by alloreactive CD4 T cells, being related to long term inhibitory effects.

## 8. The Regulation of HLA-G Expression in Pregnancy and Transplantation

The genetic regulation of HLA-G expression differs from classical HLA class I molecules: the cis-regulatory elements that are present in the proximal promoter region of classical HLA class-I genes are altered in the HLA-G promoter region [156]. Upstream transcription initiation site three* ras* response elements [157] are located along the HLA-G 1.4 kb promoter region downregulating HLA-G after the binding of the ras responsive element binding 1 factor (RREB-1).

Evidence for epigenetic control of HLA-G is initially reported for seven different HLA-G negative tumor cell lines. The treatment of these cells with the demethylating agent 5-aza-2′-deoxycytidine results in the induction of HLA-G expression [158]. In general histone acetylation is usually associated with a relaxed chromatin structure and greater levels of gene expression. Consequently, HLA-G expressing cell lines revealed enhanced levels of histone acetylation in the HLA-G promoter chromatin having been reported to be significantly enhanced, whereas in HLA-G negative cell lines histones appeared to be hypomethylated [159–161].

Moreover, the 3′UTR region of* HLA-G* is suggested to be target for certain micro RNAs (miRNA), which results in degradation of the HLA-G mRNA. The binding of miR-148a and miR-152 downregulates the expression of HLA-G by RNA degradation or translation suppression. Consequently, the ILT2 recognition and suppression of NK cell mediated killing is affected [162].

Regarding pregnancy and transplantation, a novel progesterone response element has been found leading to upregulation of HLA-G expression by progesterone [163] in trophoblasts [164], mesenchymal stem cells [165], and vascular endothelial and smooth muscle cells. Based on the latter observation progesterone is suggested to be a therapeutic option to protect against rejection and cardiac allograft vasculopathy after heart transplantation [166]. Interestingly, a computer search analysis has identified a putative consensus of hypoxia response element being located in the HLA-G promoter region. This might explain the 16-fold transcriptional upregulation of HLA-G by hypoxia induced via the iron chelator desferrioxamine [167]. Here, the relevance for ischemia reperfusion in transplanted organs has to be further investigated.

Among all cytokines and growth factors IFNs, IL10, and TGF*β* appear primarily to be the most relevant factors influencing the HLA-G expression in an allergenic situation such as pregnancy or transplantation [168, 169]. Although the mechanistic background (for overview see [170]) is not fully clarified, HLA-G expression or the release of sHLA-G molecules are found to be upregulated in response of IFN-*α*, IFN-*β*, and IFN-*γ* by trophoblasts, blood, and tumor cells [18, 168, 171–173] as well as in response to IL-10 and TGF*β* [169, 174]. However, in any case basal transcriptional levels of HLA-G are essential for its upregulation [170]. With respect to HLA-G structures, a recent study has been evidenced that HLA-G1 and HLA-G5 dimerization are enhanced by microenvironmental factors such as IFN-*β* or -*γ* [175].

In context of transplantation, several studies indicate that immunosuppressive drugs influence the HLA-G expression: steroids such as dexamethasone and hydrocortisone upregulate the HLA-G transcription and protein expression [176–178]. In heart transplant patients the antiproliferative drug everolimus but not mycophenolate (MMF) has been associated to high sHLA-G levels. The calcineurin inhibitor cyclosporine (CSA) does not show any effect on HLA-G expression [179]. The treatment with Belatacept results in an increase of sHLA-G levels. In vitro experiments have revealed that dendritic cells in the presence of Belatacept are able to secrete HLA-G, suggesting that HLA-G is involved in allograft acceptance [180]. Lastly, the antiproliferative agents Rapamycin induce ILT4 expression on DC and the release of sHLA-G molecules. These effects have been associated with an increase in the number of regulatory T cells and a shift of cytokine towards Th2 [181, 182].

## 9. How Does HLA-G Offer Novel Therapeutic Perspectives in Transplantation?

The in vitro and in vivo data clearly demonstrate that HLA-G is involved in the development of immune tolerance against allogeneic antigens, which can favour a successful transplantation. Thus, the next step should be to establish the therapeutic role of HLA-G. To this end functionally active HLA-G structures have to be produced compatible with good manufacturing practice (GMP) production conditions.

**Figure 2:**
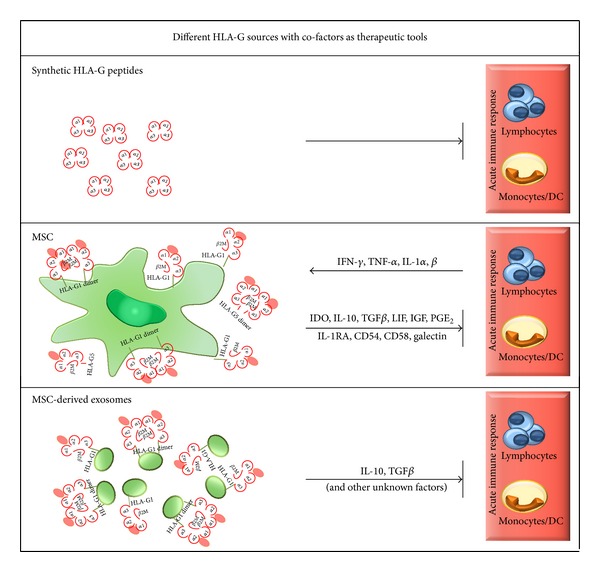
Different HLA-G sources as therapeutic tools. Either synthetic HLA-G peptides or HLA-G positive mesenchymal stem cells (MSC) and MSC-derived exosomes can be used as source to suppress acute immune response of lymphocytes and monocytes/dendritic cells (DC).

In a recent pilot study, two dimeric synthetic peptides have been engineered as therapeutic molecules. These peptides include either the alpha-3 domain of HLA-G alone or in combination with the alpha-1 domain. Both types of peptides are recognized by the ILT4 receptor but only the dimeric alpha-1/alpha-3 peptide has been found to be functional in vitro and in vivo active (Figure 2): one treatment of skin allograft recipient mice was sufficient to prolong graft survival, and four weekly treatments induce complete tolerance. In vitro experiments reveal that this peptide is able to inhibit the proliferation comparable to the dimeric full length HLA-G structure [183].

Another approach appears attractive and promising (Figure 2): to combine the function of HLA-G with the immune modulatory function of multipotent mesenchymal stem/stromal cells (MSCs). MSCs can be isolated from different tissues including bone marrow (BM), umbilical cord blood, adipose tissue, liver, and muscle. MSC has the ability to differentiate into osteoblasts, chondroblasts, or adipocytes in vitro and in vivo [184]. MSCs support the generation of damaged tissue by various factors including growth factors, cytokines and antioxidants [185]. In addition* MSCs* provide a broad complex spectrum to impair the innate and adaptive immune system. These immune modulatory functions of MSCs can be attributed to the secretion of soluble factors like indoleamine 2,3 dioxygenase (IDO), IL10, TGF*β*, prostaglandin E2 (PEG_2_), interleukin-1 receptor antagonist (IL-1RA), hepatocyte growth factor (HGF), insulin-like growth factor (IGF), leukemia inhibitory factor (LIF), and HLA-G5. The membrane expression of CD54, CD58, and HLA-G1 favours the generation and expansion of regulatory T cells (for review see [186, 187]). HLA-G/sHLA-G expressed by MSCs participates in the inhibition of T cell proliferation in allogeneic situation and in the suppression of NK cell lysis via the KIR2DL4. Secretion of HLA-5 is boosted by the direct cell contact of MSCs and allogenic T cells. MSC supernatant containing HLA-G5 and IL-10 together are able to expand FoxP3 positive regulatory T cells [188]. Based on the pioneer study by Le Blanc et al. [189] MSCs have been suggested as a novel strategy to treat severe steroid-resistant grade IV acute GvHD patients. Although there is an overall concordance that the application of MSC is in principle safe, beneficial effects have been observed only in a portion of studies [190]. Thus, a good marker is required to ensure and improve the immune modulatory functions of MSCs. Here, HLA-G may serve as a candidate marker either to predict MSC functions or to isolate and expand specifically MSCs with strong immune suppressive properties. Interestingly, MCS are able to release immunological active extracellular vesicles such as exosomes [191]. Exosomes participate in the communication of cells and/or immune regulation (for overview see [192]). In a recent study [193] MSC-derived exosomes (Figure 2), harbouring large amount of HLA-G, IL-10 and TGF*β*, were used to treat a patient suffering from severe and therapy-refractory cutaneous and intestinal GvHD grade IV. After application the patient showed significantly improved clinical GvHD symptoms without any unwanted side-effects. Thus, this study represents the first treatment in humans, in which HLA-G with the immune modulatory function of MSC-derived exosomes has been applied. The clinical outcome clearly entitles further investigations.

## 10. Conclusion

Is HLA-G on the trail of a new panacea in allogeneic situations? If we accomplish to exploit this molecule's natural function, which prevents the embryo from being rejected while nesting in an alien surrounding, in situations like transplantation, HLA-G could bring a great improvement in reducing the burden of immunosuppressive drugs or in situations of therapy refractory alloimmune responses. Therefore mapping the different functional active structures of HLA-G and its relationship to the genetic and microenvironmental background is urgently needed to further define potential areas of application.
